# Prevalence and Associated Factors of Metabolic Syndrome among Patients with Severe Mental Illness Attending a Tertiary Hospital in Southwest Uganda

**DOI:** 10.1155/2019/1096201

**Published:** 2019-11-11

**Authors:** David Collins Agaba, Richard Migisha, Rosemary Namayanja, Godfrey Katamba, Henry Mark Lugobe, Hillary Aheisibwe, Godfrey Twesigomwe, Scholastic Ashaba

**Affiliations:** ^1^Department of Physiology, Mbarara University of Science & Technology, P.O. Box 1410, Mbarara, Uganda; ^2^Department of Physiology, St. Augustine International University, P.O. Box 88, Kampala, Uganda; ^3^Department Obstetrics & Gynaecology, Mbarara University of Science & Technology, P.O. Box 1410, Mbarara, Uganda; ^4^Department of Psychiatry, Mbarara University of Science & Technology, P.O. Box 1410, Mbarara, Uganda

## Abstract

Globally, the prevalence of metabolic syndrome (MetS) and its components which are the major cardiovascular disease (CVD) risk factors, is higher among patients with severe mental illness (SMI) compared to the general population. This is mainly due to the deleterious lifestyles characterized by physical inactivity, excessive alcohol consumption, smoking, and unhealthy diets common among patients with SMI as well as due to cardiometabolic effects of psychotropic medications. Despite these conditions being highly prevalent among patients with SMI, little attention is given to these conditions during routine reviews in the mental health clinics in most low-income countries including Uganda. The main objective of this study was to determine the prevalence and associated factors of MetS among patients with SMI at Mbarara Regional Referral Hospital (MRRH), a tertiary hospital in southwestern Uganda. Through a cross-sectional study at the mental health clinic of the hospital, we recruited 304 patients with SMI and evaluated them for MetS using the National Cholesterol Education Programme Adult Treatment Panel III (NCEP ATP III) criteria. We defined the prevalence of MetS as the proportion of patients meeting the NCEP ATP III criteria. We used logistic regression to evaluate associations between MetS and independent variables. We included a total of 302 (44.37% male, 55.63% female) patients with a diagnosis of SMI in the analysis. The prevalence of MetS was 23.51% (95% CI 18.84–28.71). At multivariable logistic regression, age >40 years and long duration of mental illness (>10 years) were significantly associated with MetS. The prevalence of MetS is high among patients with psychiatric disorders, and thus metabolic screening, especially among the high-risk groups, is critical.

## 1. Introduction

Globally, the prevalence of metabolic syndrome (MetS) and its components which are the major cardiovascular disease (CVD) risk factors among patients with severe mental illness (SMI) is higher than in the general population ranging from 25–50% [1, 2]. In fact, the prevalence of MetS and its components among individuals with SMI is approximately 1.5 to 2 times higher compared with the general population [3, 4]. The high prevalence is linked to deleterious lifestyles common among patients with SMI characterized by excessive alcohol consumption, smoking, and unhealthy diet [5, 6] in addition to obesogenic side effects of antipsychotic medication [7, 8, 9].

Patients with SMI have a significantly reduced life expectancy and a mortality rate two to three times higher compared with the general population, and it is estimated that CVD accounts for 40 to 50% of this mortality [10]. This is mainly linked to medical conditions like diabetes mellitus, hypertension, and obesity that are usually not given attention during psychiatric reviews in the mental health clinics in low-income countries [11] despite these being highly prevalent among patients with SMI. Moreover, these are risk factors for CVD that carries high morbidity and mortality [10]. Although over 30% of Ugandans have some form of mental illness [12], the prevalence of MetS among patients with SMI in Uganda is not known. The aim of this study was to determine the prevalence and associated factors of MetS among patients with SMI attending the outpatient mental health clinic at Mbarara Regional Referral Hospital (MRRH), a tertiary hospital in southwestern Uganda.

## 2. Materials and Methods

A cross-sectional study was conducted at the outpatient mental health clinic of MRRH between October 2018 and March 2019. MRRH is located 250 kilometers from Kampala in southwestern Uganda. The hospital is a public health facility that serves about five million people, mainly from 10 catchment districts of southwestern Uganda. It also serves as a teaching hospital for Mbarara University of Science and Technology (MUST) medical school. The outpatient mental health clinic is run at the psychiatric ward on Tuesday and Wednesday every week, registering an average of 100 patients per clinic and on average about 1200 new patients per year. The clinic is run by a dedicated team of 3 psychiatrists, 3 occupational therapists, 1 counselor, 2 social workers, 3 psychiatric clinical officers, 5 psychiatric nurses, and postgraduate students. We consecutively recruited all patients with severe mental illness (diagnosis confirmed using the DSM-V criteria by a psychiatrist or psychiatric clinical officer), both men and women, aged 18 years and above, who were stable (in remission phase or having recovered from an acute episode) and who attended the clinic during the study period. A patient with severe mental illness was defined as one with a diagnosis of schizophrenia, schizoaffective disorder, bipolar disorder, or recurrent major depressive disorder with the disorders causing significant disability. We excluded all patients who were unstable (those who had acute symptoms with no insight or considered unable to consent as determined by the attending clinician). We also excluded pregnant women as pregnancy would affect the interpretation of waist circumference and could potentially confound blood pressure findings in case of pregnancy-induced hypertension. Patients below 18 years of age were not enrolled for the study since their risk of MetS and CVD is generally very low. Patients with known malignancy and endocrinopathies and those with hypertension or dyslipidaemia due to known aetiologies such as vascular causes or genetic syndromes were also excluded from the study as these conditions are potential confounders of the relationship between SMI and MetS.

The sample size of 304 was computed using the Kish Leslie formula of 1965 [13] using 1.96 as the critical value at 5% level of significance, taking the prevalence of metabolic syndrome among patients with severe mental illness in sub-Saharan Africa to be 23.2%, based on a study from South Africa [14], 0.05 as a margin of error and adjusting for a nonresponse rate of 10%.

The study variables of interest included age, weight, height, abdominal circumference, blood pressure, fasting blood glucose, fasting triglyceride level (TG), low-density lipoprotein (LDL), high-density lipoprotein (HDL), and HIV status. Other variables included sex, marital status, history of smoking, residence, education level, history of alcohol use, history of smoking, psychiatric diagnosis, type of psychotropic medication, and duration on psychotropic medication. The outcome variable was metabolic syndrome.

### 2.1. Measurements

Sociodemographic and clinical data were captured using a structured questionnaire. We determined the level of physical activity based on self-reports from the participants. The weight of the participants was measured using a calibrated seca weighing scale (seca 762, GmbH & Co. KG, Hamburg, Germany) to the nearest 0.5 kg with the participant not wearing shoes and heavy clothes. The height was taken using a stadiometer to the nearest 0.5 cm with the participant standing upright with the heel, buttock, and upper back along the same vertical plane. Waist circumference was measured in cm using a measuring tape in the horizontal plane midway between the inferior margin of the ribs and the superior border of the iliac crest, the measurement being recorded at the end of normal expiration. Blood pressure was measured on the left arm in mmHg by the auscultation method using a calibrated sphygmomanometer and a stethoscope with the participant seated on a chair with his or her back supported, feet on the floor, arm supported, and cubital fossa at heart level after 5 minutes of rest. Three readings were measured at a 5-minute interval taking the first and fifth Korotkoff sounds as the systolic and diastolic blood pressure (DBP), respectively. The average of the last two systolic and diastolic measurements was taken as the mean SBP and DBP, respectively [15].

### 2.2. Laboratory Tests

The participants then had their fasting blood sugar and fasting lipid profile test done. Fasting blood sugar in mmol/l was measured using a Freestyle Optium Xceed Glucometer after at least 8 hours of fasting using capillary blood obtained by finger prick under aseptic conditions. We then drew 3 ml of venous blood from the cubital fossa after at least 8 hours of fasting under aseptic technique, and stored it in a nonheparinized vacutainer. The samples were analyzed for total cholesterol, high-density lipoprotein (HDL), triglycerides (TGs), and low-density lipoprotein (LDL) using an ELITech Group PIT-CHSL-4-v21 (09/2016) machine following the enzymatic-colorimetric method. HIV antibody testing was done according to the Uganda Ministry of Health HIV testing algorithm from the 3 ml of blood collected. HIV testing was done using two rapid tests (Alere Determine™ HIV-1/2, Abbott, Illinois, USA; HIV 1/2 STAT-PAK® Assay, Chembio Diagnostic Systems Inc, New York, USA) and a third one (SD BIOLINE HIV-1/2 3.0, Standard Diagnostics 03FK35, 03FK30 Inc., Giheung-gu, Republic of Korea) in case the first two tests were discordant following the standard national algorithm.

### 2.3. Data Handling and Analysis

The data were cleaned then double entered into Epi Data 3.1, after which they were exported to STATA version 12 (StataCorp, College Station, Texas, USA) for analysis. Descriptive analysis of independent variables was done. Continuous variables were described as mean ± SD while categorical variables were described as percentages. Student's *t*-test and chi-square test were used for continuous and categorical variables, respectively, to describe differences between participants with MetS and those without MetS. MetS were defined according to the National Cholesterol Education Programme Adult Treatment Panel III (NCEP ATP III) definition as the presence of three or more of the following criteria:

1. Abdominal obesity: waist circumference (WC) >102 cm in men and >88 cm in women
2. Hypertriglyceridemia: ≥150 mg/dl (1.695 mmol/l)
3. Low high-density lipoprotein (HDL): <40 Mg/dl in men and <50 Mg/dl in women
4. High blood pressure (BP): ≥130/85 mmHg or taking antihypertensive Medication
5. High fasting glucose: ≥110 mg/dl (6.1 mmol/l) or taking antihyperglycemic medication

Prevalence of metabolic syndrome was the proportion of participants who met the above criteria.

Binary logistic regression analysis was used to evaluate associations between independent variables and metabolic syndrome. Variables which were found significant in the binary logistic model (associated with *p* value < 0.2) were entered into a final multivariable logistic regression model to identify independent predictors of MetS among patients with SMI.

### 2.4. Ethics

Ethical clearance for the study was obtained from the Mbarara University of Science and Technology Research Ethics Committee (No. 07/08-18) and the Uganda National Council for Science and Technology (HS 2548). Data were collected by research assistants trained to handle data with confidentiality. We respected the guidelines of Helsinki and CIOMS-2002 (Council for International Organizations of Medical Sciences) regarding research with humans, avoiding any type of physical or moral damage. Written informed consent was obtained by research assistants from all participants. In addition, we sought consent from participants' caretakers.

## 3. Results

Of the 304 participants recruited, results of 302 participants were analyzed. Two participants were excluded from the analysis because they did not undergo laboratory testing for lipid profile and as a result could not be assessed for the outcome of interest. Participants' sociodemographic characteristics are presented in Table 1. The mean age of the participants was 38.5 (±13.66) years. Participants with MetS were on average significantly older than those without MetS (*p* < 0.001).

The clinical characteristics of study participants are presented in Table 2. The majority (63.58%) of the participants had a diagnosis of bipolar disorder with the majority (45.70%) having been with mental illness for less than 5 years. However, there was a significant difference (*p* < 0.001) in duration of mental illness across the two MetS diagnostic groups with majority (53.52%) of patients with MetS having been with mental illness for >10 years compared with those without MetS, majority (49.35%) of whom had had mental illness for <5 years. The majority (48.68%) of the patients had been on treatment for less than 5 years. Across the two MetS diagnostic groups, there was a statistically significant difference (*p*=0.001) in the duration of psychotropic medication with the majority (46.48%) of patients with MetS having been on treatment for 5–10 years, whereas those without MetS (51.52%) were on treatment for less than 5 years. Most of the participants (81.79%) were prescribed antipsychotic medication with most of them on typical antipsychotics (93.93%). There was no statistically significant difference in the type of medication between the two MetS diagnostic groups.

Out of the 302 participants, 71 met the National Cholesterol Education Programme Adult Treatment Panel III definition of MetS giving a prevalence of 23.51% (95% CI 18.84–28.71). The prevalence was higher in women (27.98% (95% CI 21.34–35.41)) compared with men (17.91% (95% CI 11.83–25.47)). At univariate analysis, age over 40 years, female sex, having ever married or lived with a partner, having been with mental illness for over 10 years, and being on psychotropic medication for 5–10 years were associated with MetS as shown in Tables 3 and 4.

After multivariable logistic regression, age 40–60 years aOR 3.37 (CI 1.66–6.84) and duration of mental illness longer than 10 years aOR 2.92 (CI 1.38–6.18) were identified as the independent risk factors for metabolic syndrome among patients with severe mental illness as shown in Table 5 below.

## 4. Discussion

Our results show a high prevalence of MetS of 23.51% among patients with SMI. The prevalence in this study is in keeping with rates reported in Thailand (22.8%) [16], Mexico (21%) [17], South Africa (23.2%) [14], and Spain (26.5%) [18], and it does not differ much from the global range (25%–50%) found in a meta-analysis by Vancampfort and colleagues [2]. This prevalence is however much lower than that found in studies conducted in Australian (54%) [19], Hong Kong (35%) [20], England (57%) [21], and the US (49.2%) [4]. This difference may be explained by the fact that these studies enrolled patients majorly from urban areas of high-income countries who were mainly on atypical antipsychotic medications that are more associated with MetS compared to our patients of whom the majority was from rural areas (74.5%) and on typical antipsychotic medications (93.93%). The prevalence of MetS was higher among females (27.98%) compared with males (17.91%). Similar findings have been reported in several other studies [14, 22, 23]. The difference could be attributed to different cutoff points based on sex, set as criteria for MetS like waist circumference and high-density lipoproteins [24]. However, it is important to note that some studies have found no difference in the prevalence of MetS based on gender [25, 26, 27].

From our study, age above 40 years and has had a mental illness for more than 10 years were independently associated with MetS. The association of age with MetS that we found in this study is in keeping with that reported in the previous research literature [26, 28, 29]. This is because many conditions which increase in prevalence with increasing age, such as obesity, insulin resistance, inflammation, changes in the activity of the hypothalamus-hypophysis suprarenal axis, and stress and hypertension, also contribute to increase in the prevalence of MetS [30]. Our study also found that a longer duration of mental illness is a major risk factor for MetS. This finding is in agreement with two meta-analyses which found that the duration of psychiatric illness which is a proxy for the duration of exposure to psychotropic medication is the strongest factor influencing the development of MetS [26, 31]. The longer the duration of treatment, the higher the risk for MetS since psychotropic medications cause obesity [8, 32, 33, 34, 35] through an increase in appetite for food with limited activity. Obesity leads to insulin resistance and then diabetes mellitus [36, 37], and the two conditions predispose to atherosclerosis and then HTN which are all components of MetS. This study found no association between MetS and sex, contrary to findings from several studies that have found an increased risk for MetS in women compared with men [14, 38, 39]. Studies have found that women are more likely to be obese yet obesity is a major risk factor for MetS [40]. One study also found that women have increased susceptibility to metabolic side effects of antipsychotic medication [41]. This study found no association between metabolic syndrome and psychotropic medication probably because majority of the patients in our study were on older generation psychotropic medication compared with studies which found these associations in which patients were majorly on newer generation antipsychotics, antidepressants, and mood stabilizers [42, 43, 44].

We did not find an association between MetS and HIV status yet HIV is associated with dysregulated inflammatory responses through suppressing genes that control inflammation [45]; and this inflammatory environment along with higher white blood cell counts act as the metabolic risk factor for MetS [46]. In addition, highly active antiretroviral treatment (HAART) has been found to cause insulin resistance, hyperlipidemia, and lipodystrophy [47]. We, however, found a higher prevalence of HIV (11.26%) in these participants, a prevalence almost twice the 6.0% found among adults aged 15–64 years in the Ugandan general population [48, 49]. This calls for interventions to address this discrepancy in HIV prevalence among patients with SMI.

Our study had limitations worth mentioning. It was conducted in one hospital, and findings from the study may not be generalizable beyond the population of patients attending MRRH. The study may have been subjected to social acceptability bias from participants wanting to provide socially desirable responses to questions about risky lifestyles of patients with severe mental illness such as smoking and alcohol consumption. We did not assess the diet and dietary habits of the participants yet these are important determinants of MetS. The study design was cross sectional, which is not the most suitable method for making causal inferences.

## 5. Conclusion

The prevalence of metabolic syndrome among patients with SMI attending Mbarara Regional Referral Hospital is high and is associated with age and long duration of mental illness. Therefore, metabolic screening, especially among patients aged 40 years and above and on treatment for 10 years or more, is critical. There is also a need for health education to patients with SMI about lifestyle modification through exercise, diet, and ceasing smoking and alcohol consumption as preventative measures for MetS.

## Figures and Tables

**Table 1:** Sociodemographic characteristics of participants by MetS status.

Characteristic						Overall *N* = 302	MetS (*N* = 71)	No MetS (*N* = 231)	*p* value
						*n*/*N* (%)	*n*/*N* (%)	*n*/*N* (%)	
Age in years (mean ± SD)						38.5 (±13.66)	45.73 (±12.45)	36.47 (±13.30)	<0.001^*∗*^

Sex									0.040^*∗*^
Male						134 (44.37)	24 (33.80)	110 (47.62)	
Female						168 (55.63)	47 (66.20)	121 (52.38)	

Marital status									0.008^*∗*^
Single						115 (38.08)	16 (22.54)	99 (42.86)	
Married/has a partner						121 (40.07)	37 (52.11)	84 (36.36)	
Divorced/separated/died						66 (21.85)	18 (25.35)	48 (20.78)	

Education level									0.727
Never attended						34 (11.26)	09 (12.68)	25 (10.82)	
≤Secondary						199 (65.89)	44 (61.97)	155 (67.10)	
Tertiary/university						69 (22.85)	18 (25.35)	51 (22.08)	

Employment									0.825
Unemployed						45 (14.90)	10 (14.08)	35 (15.15)	
Employed						257 (85.10)	61 (85.92)	196 (84.85)	

Area of residence									0.057
Rural						225 (74.50)	59 (83.10)	166 (71.86)	
Urban						77 (25.50)	12 (16.90)	65 (28.14)	

Ever smoked									0.633
No						272 (90.07)	65 (91.55)	207 (89.61)	
Yes						30 (9.93)	06 (8.45)	24 (10.39)	

Ever taken alcohol									0.929
No						197 (65.23)	46 (64.79)	151 (65.37)	
Yes						105 (34.77)	25 (35.21)	80 (34.63)	

*Note.* MetS: metabolic syndrome; ^*∗*^statistically significant.

**Table 2:** Clinical characteristics of participants as per MetS status.

Characteristic	Overall *N* = 302	MetS (*N* = 71)	No MetS (*N* = 231)	*p*-value
	*n*/*N* (%)	*n*/*N* (%)	*n*/*N* (%)	
HIV status				0.998
Negative	268 (88.74)	63 (88.73)	205 (88.74)	
Positive	34 (11.26)	8 (11.27)	26 (11.26)	
Weight, kg (mean ± SD)	68.18 (±14.14)	79.86 (±13.72)	64.73 (±12.24)	<0.001^*∗*^

Mental illness				0.971
BAD	192 (63.58)	46 (64.79)	146 (63.20)	
Schizophrenia	79 (26.16)	18 (25.35)	61 (26.41)	
Depression	31 (10.26)	07 (9.86)	24 (10.39)	

Duration of mental illness (years)				<0.001^*∗*^
<5	138 (45.70)	24 (33.80)	114 (49.35)	
5–10	68 (22.52)	09 (12.68)	59 (25.54)	
>10	96 (31.79)	38 (53.52)	58 (25.11)	

Duration of psychotropic medication (years)				0.001^*∗*^
<5	147 (48.68)	28 (39.44)	119 (51.52)	
5–10	88 (29.14)	33 (46.48)	55 (23.81)	
>10	67 (22.19)	10 (14.08)	57 (24.68)	

Antipsychotic medication	247 (81.79)	56 (78.87)	191(82.68)	0.467

Antipsychotic class (*N* = 247)				0.098
Typical	232 (93.93)	50 (89.29)	182 (95.29)	
Atypical	15 (6.07)	06 (10.71)	09 (4.71)	
Mood stabilizer	161 (53.31)	34 (47.89)	127 (54.98)	0.295
Antidepressant	56 (18.54)	18 (25.35)	38 (16.45)	0.091

*Note.* BAD: bipolar disorder; MetS: metabolic syndrome; hx: history; ^*∗*^statistically significant.

**Table 3:** Sociodemographic factors associated with MetS at bivariate analysis.

Characteristic	% of MetS	Bivariate analysis	*p* value
	*n*/*N* (%)	Or (95% CI)	
Age category (years)			
<40	21 (29.58)	Ref	
40–60	43 (60.56)	4.55 (2.51–8.24)	<0.001^*∗*^
>60	07 (9.86)	4.19 (1.49–11.84)	0.007^*∗*^

Sex			
Male	24 (33.80)	Ref	
Female	47 (66.20)	1.78 (1.02–3.10)	0.042^*∗*^

Marital status			
Single	16 (22.54)	Ref	
Married/lived with a partner	37 (52.11)	2.73 (1.41–5.24)	0.003^*∗*^
Divorced/separated/died	18 (25.35)	2.32 (1.09–4.94)	0.029^*∗*^

Employment			
Unemployed	10 (14.08)	Ref	
Employed	61 (85.92)	1.09 (0.51–2.33)	0.825

Area of residence			
Rural	59 (83.10)	Ref	
Urban	12 (16.90)	0.52 (0.26–1.03)	0.06

Ever smoked			
No	65 (91.55)	Ref	
Yes	06 (8.45)	0.80 (0.31–2.03)	0.633

Ever taken alcohol			
No	46 (64.79)	Ref	
Yes	25 (35.21)	1.03 (0.58–1.79)	0.929

*Note.* MetS: metabolic syndrome; OR: odds ratio; CI: confidence interval; Ref: reference category.

**Table 4:** Clinical factors associated with MetS at bivariate analysis.

Characteristic	% of MetS	Bivariate analysis	*p* value
	*n*/*N* (%)	OR (95% CI)	
HIV status			
Negative	63 (88.73)	Ref	
Positive	08 (11.27)	1.00 (0.43–2.32)	0.998

Mental illness			
BAD	46 (64.79)	Ref	
Schizophrenia	18 (25.35)	0.94 (0.50–1.74)	0.836
Depression	07 (9.86)	0.93 (0.37–2.29)	0.867

Duration of mental illness (years)			
<5	24 (33.80)	Ref	
5–10	09 (12.68)	0.72 (0.32–1.66)	0.446
>10	38 (53.52)	3.11 (1.71–5.68)	<0.001^*∗*^

Duration of psychotropic medication (years)			
<5	28 (39.44)	Ref	
5–10	33 (46.48)	2.55 (1.40–4.62)	0.002^*∗*^
>10	10 (14.08)	0.75 (0.34–1.64)	0.465

Antipsychotic			
No	15 (21.13)	Ref	
Yes	56 (78.87)	0.78 (0.40–1.52)	0.468

Antipsychotic class			
Typical	50 (89.29)	Ref	
Atypical	06 (10.71)	2.42 (0.82–7.14)	0.107

Mood stabilizer			
No	37 (52.11)	Ref	
Yes	34 (47.89)	0.75 (0.44–1.28)	0.296

Antidepressant			
No	53 (74.65)	Ref	
Yes	18 (25.35)	1.72 (0.91–3.26)	0.094

Level of physical activity			
<150 min of moderate exercise or 75 min of vigorous/ week	65 (91.55)	Ref	
≥150 min of moderate exercise or 75 min of vigorous/ week	06 (8.45)	0.80 (0.31–2.03)	0.633

*Note.* BAD: bipolar disorder; MetS: metabolic syndrome; hx: history; OR: odds ratio; CI: confidence interval; Ref: reference category.

**Table 5:** Factors associated with MetS at multivariable logistic regression.

Characteristic	Multivariable analysis	*p* value
	Adjusted OR (95% CI)	
Age category (years)		
<40	Ref	
40–60	3.37 (1.66–6.84)	0.001^*∗*^
>60	2.41 (0.62–9.35)	0.203

Sex		
Male	Ref	
Female	1.87 (0.94–3.71)	0.074

Duration of mental illness (years)		
<5	Ref	
5–10	0.93 (0.34–2.54)	0.891
>10	2.92 (1.38–6.18)	0.005^*∗*^

Antipsychotic class		
Typical	Ref	
Atypical antipsychotic	2.53 (0.78–8.19)	0.121

*Note.* MetS: metabolic syndrome; OR: odds ratio; CI: confidence interval; Ref: reference category; ^*∗*^ *=* statistically significant.

## Data Availability

The data used to support the findings of this study are available on request from the corresponding author.
